# ATM Inhibition-Induced ISG15/IFI27/OASL Is Correlated with Immunotherapy Response and Inflamed Immunophenotype

**DOI:** 10.3390/cells12091288

**Published:** 2023-04-30

**Authors:** Chi-Han Huang, Yun-Cian Huang, Jun-Kai Xu, Si-Yun Chen, Lu-Chia Tseng, Jau-Ling Huang, Chang-Shen Lin

**Affiliations:** 1 Graduate Institute of Medicine, College of Medicine, Kaohsiung Medical University, Kaohsiung 807, Taiwan; cheryl60286@gmail.com (C.-H.H.); yuncian1103@gmail.com (Y.-C.H.); qoxoruby@gmail.com (S.-Y.C.); 2 Department of Bioscience Technology, College of Health Science, Chang Jung Christian University, Tainan 711, Taiwan; 109b22503@mailst.cjcu.edu.tw (J.-K.X.); luke20000820@gmail.com (L.-C.T.); 3 Department of Medical Research, Kaohsiung Medical University Hospital, Kaohsiung Medical University, Kaohsiung 807, Taiwan; 4 Department of Biological Sciences, National Sun Yat-Sen University, Kaohsiung 804, Taiwan

**Keywords:** ATM, interferon-stimulated gene, immune checkpoint blockade, immunotherapy, tumor microenvironment, cisplatin resistance, oral cancer

## Abstract

Immune checkpoint blockade (ICB) therapy can improve the survival of cancer patients with a high tumor mutation burden (TMB-H) or deficiency in DNA mismatch repair (dMMR) in their tumors. However, most cancer patients without TMB-H and dMMR do not benefit from ICB therapy. The inhibition of ATM can increase DNA damage and activate the interferon response, thus modulating the tumor immune microenvironment (TIME) and the efficacy of ICB therapy. In this study, we showed that ATM inhibition activated interferon signaling and induced interferon-stimulated genes (ISGs) in cisplatin-resistant and parent cancer cells. The ISGs induced by ATM inhibition were correlated with survival in cancer patients who received ICB therapy. In oral cancer, high expressions of *ISG15*, *IFI27*, and *OASL* were associated with low expressions of *ATM*, the activation of inflamed immune pathways, and increased tumor-infiltrating scores of CD8+ T, natural killer, and dendritic cells. The high expressions of *ISG15*, *IFI27*, and *OASL* were also correlated with complete remission in patients with cervical cancer treated with cisplatin. These results suggest that ATM inhibition can induce the interferon response and inflamed TIME, which may benefit ICB therapy.

## 1. Introduction

Oral cancer consists of malignancies of the lip, tongue, buccal mucosa, mouth floor, hard palate, and gum. It is estimated that 377,713 new cases of oral cancer and 177,757 new deaths from oral cancer are found worldwide in 2020 [1]. Cigarette smoking, alcohol consumption, chewing of betel quid, and infection with human papillomavirus are risk factors for the development of oral cancer [2,3]. In addition to surgical resection, oral cancer can be treated with radiation therapy, photodynamic therapy, chemotherapy, and targeted therapy using synthetic small molecule drugs, biodrugs, and immunotherapy [4,5,6]. However, 5-year survival rates for patients with oral cancer remain between 50 and 66% [7]. Therefore, novel approaches are needed to improve the survival rates of patients with oral cancer.

Immune checkpoint blockade (ICB) therapy can improve patient survival by activating the anticancer immune response in the tumor immune microenvironment (TIME) [8]. However, the benefit of ICB therapy is restricted to a subset of cancer patients, whose cancer cells usually exhibit a high tumor mutation burden (TMB-H), mismatch repair deficiency (dMMR), or high microsatellite instability (MSI-H) [9,10,11,12,13,14]. Cancer cells with characteristics of TMB-H, dMMR, or MSI-H can generate more neoantigens, and then activate the immune response and sculpt the “hot” TIME, which contributes to ICB therapy. In contrast, cancer patients without these characteristics usually have a “cold” tumor and may not respond well to ICB therapy [8].

To overcome the poor response of ICB therapy in cancer patients with cold tumors, the inactivation of the DNA damage response (DDR) is a potential strategy to elicit the hot TIME, and therefore can improve the efficacy of ICB therapy [15,16,17,18]. For example, the poly (ADP-ribose) polymerase (PARP) inhibitor (PARPi) olaparib can activate the interferon response in mouse lung cancer models, leading to elevated expressions of CXCL10 and CCL5 and increased infiltration of cytotoxic CD8+ T cells in the TIME. As a result, olaparib is capable of increasing the efficacy of ICB therapy [19].

Interferon signaling plays a crucial role in the regulation of the immune response, including the responsiveness of ICB therapy [20]. It has been demonstrated that DDR inhibition-induced DNA damage can induce an interferon response through cGAS-mediated DNA sensing in cancer cells, leading to the activation of the STING-TBK1-IRF3 axis and then induction of type I interferons and interferon-stimulated genes (ISGs) [21]. Type I interferons and many ISGs can attract and activate innate and adaptive immune cells, including dendritic cells (DCs), natural killer (NK) cells, CD4+ T-helper cells, and cytotoxic CD8+ T cells [17,20]. Consequently, DDR inhibition in cancer cells can create the “hot” TIME and improve ICB therapy.

In addition to Olaparib, the ATM inhibitor (ATMi) can also induce the interferon response and sensitize cancer cells to ICB in preclinical models [22,23,24]. Furthermore, *ATM* gene mutation is found to be correlated with “hot” TIME and increased tumor infiltration of cytotoxic lymphocytes in human endometrial cancers [25]. Among the 1661 patients who received ICB therapy (the MSK-ICB cohort) [26], an improved overall survival (OS) was observed in patients with *ATM* gene mutation compared to those with wild-type *ATM* genes [22]. Pan et al. also reported that bladder cancer patients with *ATM* gene mutation are classified into a “low-risk” group based on the responsiveness of ICB therapy, which also predicts patients’ OS [27]. These results suggest that *ATM* gene mutation or ATM inhibition may contribute to the “hot” TIME and have the potential to improve the efficacy of ICB therapy.

Although ATM inhibition-elicited interferon response is demonstrated in some cancer cells, it is unclear whether this immune activation can be found in oral cancer or cisplatin-resistant (CDDP-R) cancers. Here, we found that the ATMi KU55933 could activate the interferon response and ISG expressions in CDDP-R and parent cancer cells. The high expressions of KU55933-induced ISGs were correlated with a good OS rate in cancer patients who received ICB therapy. Among these ISGs, the high expressions of *ISG15*, *IFI27*, and *OASL* were associated with low *ATM* expression, the activation of immune pathways, and high tumor-infiltrating scores of DCs, NK, and cytotoxic CD8+ T cells in oral cancer. These results suggest that ATM inhibition is able to induce the interferon response in CDDP-R cancers and that the high-expression of *ISG15*/*IFI27*/*OASL* is correlated with inflamed TIME in oral cancer, which is indicative of a better response to ICB therapy.

## 2. Materials and Methods

### 2.1. Study Design

**Figure 1:**
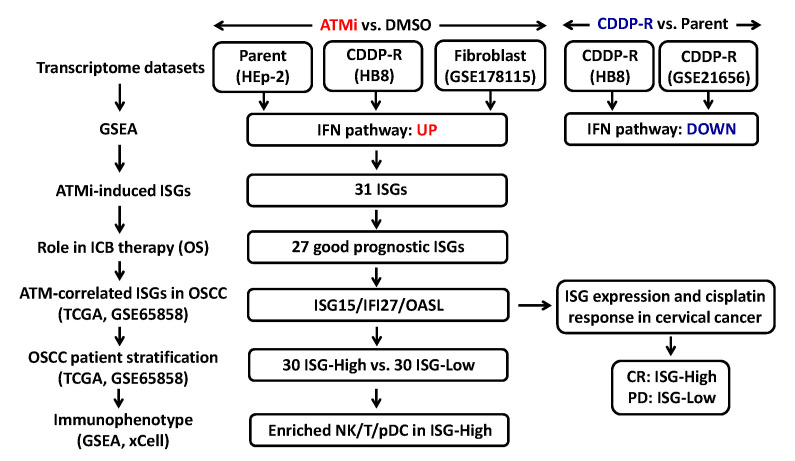
The study pipeline. ATMi, ATM inhibitor. CDDP-R, cisplatin-resistant. GSEA, gene set enrichment analysis. IFN, interferon. ISGs, interferon-stimulated genes. ICB, immune checkpoint blockade. OS, overall survival. OSCC, oral squamous cell carcinoma. TCGA, the Cancer Genome Atlas. xCell, an algorithm for cell-type enrichment analysis. NK, natural killer cells. pDC, plasmacytoid dendritic cells. CR, complete remission. PD, progression disease.

Figure 1 shows the study pipeline. Gene expression profiles of ATMi-treated CDDP-R and parent cancer cells and normal human dermal fibroblasts (GSE178115) were applied to gene set enrichment analysis (GSEA) to find the pathways that were significantly affected by ATM inhibition, resulting in upregulation of interferon pathways. In contrast, interferon pathways were downregulated in CDDP-R cancer cells compared to their parent cancer cells. To explore the roles of ATMi-induced ISGs in ICB therapy, upregulated ISGs were intersected in ATMi-treated CDDP-R and parent cancer cells to obtain a collection of 31 ISGs, whose expressions on OS rate of cancer patients with ICB therapy were examined by Kaplan–Meier survival analysis. The high expressions of 27 ISGs were correlated with improved OS rates and, among them, the high expressions of *ISG15*, *IFI27*, and *OASL* were significantly associated with low *ATM* expression (mimicry of ATM inhibition) in two oral cancer data sets (TCGA and GSE65858). The oral cancer patients were then stratified into subgroups of “High” and “Low” expression based on the joint expression of *ISG15*/*IFI27*/*OASL* to compare their immunophenotype using GSEA and xCell: a cell-type enrichment analysis algorithm. Meanwhile, the roles of *ISG15*, *IFI27*, and *OASL* expression in the cisplatin response were explored in the cervical cancer cohort (TCGA-CESC).

### 2.2. Cell Culture and Establishment of Cisplatin-Resistant Cancer Cells

The squamous cell carcinoma cell line HEp-2 and its derived cisplatin-resistant cell subclone HB8 (IC_50_: 33.5 μM) were described in our previous paper [28]. The oral cancer cells, CAL27-derived cisplatin-resistant cell subclone CAL-A8 (IC_50_: 20.5 μM), were obtained by chronic selection in medium containing increasing doses of cisplatin for > 3 months. All cells were cultured in Dulbecco’s modified Eagle’s medium (HyClone, Logan, UT, USA) and 10% fetal bovine serum (Invitrogen, Carlsbad, CA, USA) at 37 °C and 5% CO_2_. The cisplatin-resistant cells were kept in medium containing 2 μM cisplatin, and were shifted in normal medium without cisplatin for 3 days before KU55933 treatment and RNA extraction. KU55933 and cisplatin were purchased from BioVision (Mountain View, CA, USA) and Sigma (St. Louis, MO, USA), respectively.

### 2.3. Transcriptomic Data Sets and Gene Set Enrichment Analysis (GSEA)

Total RNA from KU55933-treated HEp-2 (10 μM, 24 h) and HB8 (10 and 20 μM, 24 h) cells was harvested using Tri-reagent (Sigma) for the analysis of gene expression profiles (Human OneArray Plus HOA 7.1, Phalanx Biotech, Taiwan), as described in our paper [29]. Transcriptome data sets of normal human dermal fibroblasts treated with KU60019 and lung cancer cells resistant to cisplatin were retrieved from NCBI GEO with accession numbers GSE178115 and GSE21656, respectively. The transcriptome data sets of oral cancer specimens of TCGA and GSE65858 were as described in our previous paper [30]. Clinical (cisplatin response) and transcriptome data from cervical cancer (TCGA-CESC cohort) were downloaded from UCSC Xena on 14 June 2022. These transcriptomic data were analyzed using GSEA version 4.1 with collections of Hallmark and GO biological process (GOBP) gene set in the Molecular Signatures Database version 7.1 [31].

### 2.4. Real-Time Quantitative PCR (RT-qPCR)

Total RNA was converted to cDNA using the High-Capacity cDNA Archive Kit (Applied Biosystems, Foster City, CA, USA). The resulting cDNA was used for qPCR in the StepOne System (Applied Biosystems) as described [29]. The qPCR results were examined by dissociation (melting) curve analysis to ensure the specificity of qPCR products. Each sample was run for target genes and *GAPDH*, which served as an internal control. Relative gene expression was calculated by 2^−ΔΔC^_T_ method using *GAPDH* as an internal control. Results were represented as mean ± standard deviation (*n* = 3–4). The primer sequences are shown in Table 1.

### 2.5. Kaplan–Meier Survival Analysis

The Kaplan–Meier OS plots were conducted using the Kaplan–Meier plotter immunotherapy database (https://kmplot.com, accessed on 30 November 2022) [32] according to ATM and ISG expressions. The best cut-off values were determined by ROC curve analysis.

### 2.6. Cell-Type Enrichment Analysis

The transcriptome data of the ISG15/IFI27/OASL-high (*n* = 30) and -low (*n* = 30) subgroups of oral cancer cohorts of TCGA and GSE65858 were analyzed using the xCell algorithm [33] to obtain enrichment scores for various types of immune cells. Differences in cell-type enrichment scores between the ISG15/IFI27/OASL-high and -low subgroups were examined using the Mann–Whitney U test.

## 3. Results

### 3.1. The Interferon Response Was Activated upon ATM Inhibition but Was Repressed in Cisplatin-Resistant (CDDP-R) Cancer Cells

**Figure 2:**
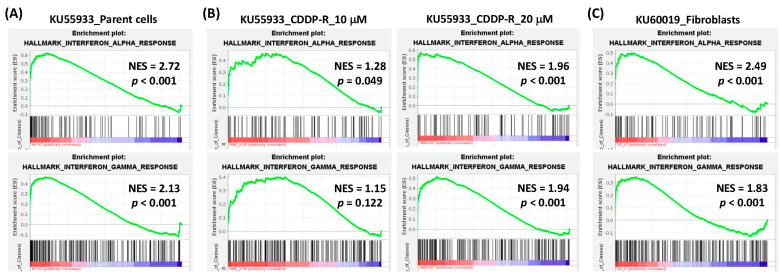
Activation of interferon pathways by ATM inhibitors in cancer cells and normal fibroblasts. The enrichment plots of interferon-α (**upper** panels) and interferon-γ (**lower** panels) pathways in the KU55933 (10 μM)-treated (versus vehicle control) parent cancer cells (**A**), cisplatin-resistant (CDDP-R) cancer cells (**B**), and KU60019-treated normal human dermal fibroblasts ((**C**), GSE178115). NES, normalized enrichment score (positive values stand for activation). The red and blue colors represent positive and negative correlation with the treatments of ATM inhibitors, respectively.

**Figure 3:**
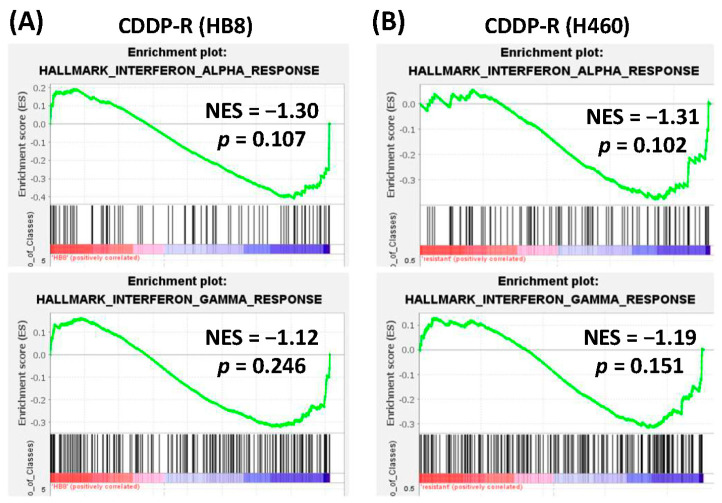
Suppression of interferon pathways in cisplatin-resistant (CDDP-R) cancer cells. The enrichment plots of interferon-α (**upper** panels) and interferon-γ (**lower** panels) pathways in cisplatin-resistant (CDDP-R) HB8 (**A**) and H460 ((**B**), GSE21656) cancer cells (versus parent cancer cells). NES, normalized enrichment score (negative values stand for inhibition). The red and blue colors represent positive and negative correlation with CDDP-R, respectively.

Previously, we found that the ATM inhibitor (ATMi) KU55933 activated interferon pathways in cancer cells (Figure 2A) [29]. Here, gene expression profiling and GSEA demonstrated that KU55933 also induced the interferon response in CDDP-R cancer cells (Figure 2B). Additionally, another ATMi KU60019 was found to activate interferon pathways in normal human dermal fibroblasts (GSE178115, Figure 2C). These results suggest that ATM inhibition can activate the interferon response in both normal and cancer cells, including CDDP-R cancer cells. In contrast, interferon pathways tended to be downregulated in CDDP-R cancer cells compared to the parent cancer cells (Figure 3A). The suppression of the interferon response was also observed in another CDDP-R lung cancer data set (GSE21656, Figure 3B). These results implied that CDDP-R cancer cells were able to evade interferon-mediated immune responses, which could be reactivated through the inhibition of ATM kinase in CDDP-R cancer cells.

### 3.2. The KU55933-Induced Interferon-Stimulated Genes (ISGs) Were Associated with Overall Survival (OS) in Cancer Patients with ICB Therapy

**Figure 4:**
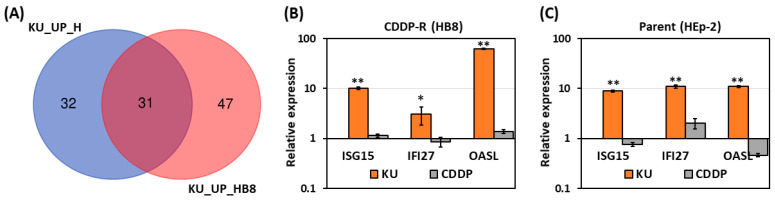
Upregulation of interferon-stimulated genes by ATM inhibitor. (**A**) Intersection of KU55933-induced ISGs in parent (**left** circle, 63 ISGs) and cisplatin-resistant (**right** circle, 78 ISGs) cancer cells. (**B**,**C**). The gene expressions of ISG15, IFI27, and OASL in cisplatin-resistant (**B**) and parent (**C**) cancer cells treated with KU55933 (KU) or cisplatin (CDDP) were examined by RT-qPCR. * *p* < 0.05 versus control. ** *p* < 0.001 versus control, Student’s *t*-test.

Because interferon signaling plays a crucial role in modulating cancer immunotherapy [20], we next aimed to identify the ISGs that were upregulated by ATM inhibition and implicated in ICB therapy. First, the significantly upregulated ISGs were intersected in KU55933-treated CDDP-R and parent cancer cells, resulting in 31 ISGs (Figure 4A). The RT-qPCR results confirmed that the expressions of *ISG15*, *IFI27*, and *OASL* increased significantly in KU55933-treated CDDP-R (Figure 4B) and parent (Figure 4C) cancer cells. In contrast, cisplatin treatment did not apparently induce the expressions of these ISGs in both CDDP-R and parent cancer cells (Figure 4B,C). The induction of *ISG15*, *IFI27*, and *OASL* expression by KU55933 was also observed in oral cancer cells CAL27 and derived CDDP-R cancer cells (Supplementary Figure S1). The results of RT-qPCR verified that KU55933 significantly upregulated additional ISGs including *ISG20*, *DDX58*, *DDX60*, *CCL5*, and *IFIT3* in CDDP-R cancer cells (Supplementary Figure S2). These results demonstrate that ATM inhibition can induce the expression of several ISGs in cancer cells, including CDDP-R cancer cells. On the other hand, the expression levels of *ISG15*, *IFI27*, *OASL*, *ISG20*, and *DDX58* were lower in CDDP-R cancer cells than in parent cancer cells (Supplementary Figure S3), which were consistent with the transcriptome profiling data (Figure 3).

**Figure 5:**
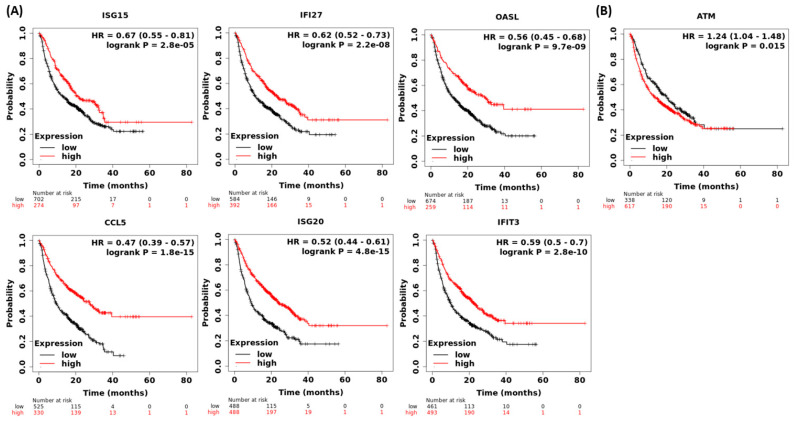
Kaplan–Meier plots of overall survival of cancer patients who received immune checkpoint blockade therapy. The plots were computed based on the expressions of *ISG15*, *IFI27*, *OASL*, *CCL5*, *ISG20*, *IFIT3*, (**A**) and *ATM* (**B**) in the Kaplan–Meier plotter immunotherapy database (https://kmplot.com, accessed on 30 November 2022). The best cut-off values were determined by ROC curve analysis.

Next, the roles of KU55933-induced ISGs in the survival of cancer patients who received ICB therapy were examined using the Kaplan–Meier plotter, a web-based tool that can analyze the transcriptome and survival data from multiple types of cancer. The results showed that high expressions of *ISG15*, *IFI27*, *OASL*, *CCL5*, *ISG20*, *IFIT3*, (Figure 5A) and other 21 ISGs (Supplementary Figure S4) were significantly correlated with improved OS rates in cancer patients receiving ICB therapy. In particular, low *ATM* expression, which might exhibit a phenotype similar to that of ATM inhibition, was associated with better OS in cancer patients treated with ICB (Figure 5B). In addition, Hu et al. reported that cancer patients treated with ICB and with the *ATM* gene mutation exhibited higher OS rates than those with the wild-type *ATM* gene [22]. These results suggest that ATM inhibition and high expressions of certain ISGs are beneficial for cancer patients who receive ICB therapy.

### 3.3. ATM and ISG15/IFI27/OASL Expression Levels Were Inversely Correlated in Oral Cancer

**Figure 6:**
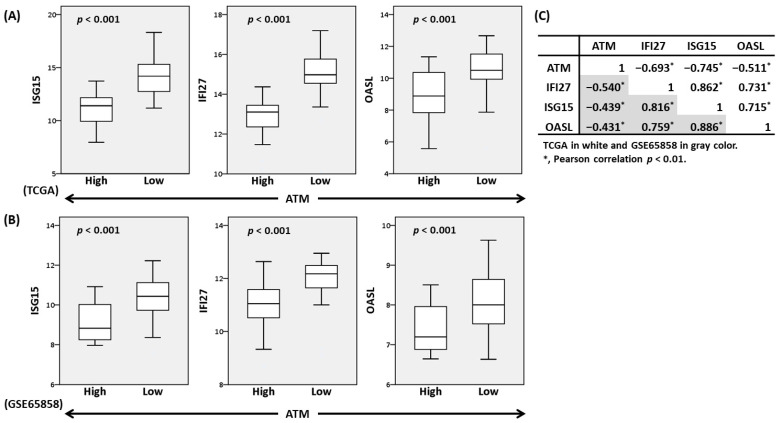
The inverse correlation of *ATM* and ISG expressions in oral cancer. (**A**,**B**) The box plots show the expressions of *ISG15*, *IFI27*, and *OASL* in the *ATM*-high (*n* = 30) and *ATM*-low (*n* = 30) subgroups of the oral cancer data sets of TCGA (**A**) and GSE65858 (**B**). *p*, Mann–Whitney U test. (**C**) The Pearson correlation coefficient matrix of gene expressions between *ATM* and the three ISGs.

Although the United States Food and Drug Administration has approved the use of ICB therapy for patients with head and neck cancer [8], the influence of *ATM* and ISG expression on the TIME and efficacy of ICB in oral cancer is unclear. To examine the roles of *ATM* and KU55933-induced ISGs in oral cancer TIME, patients from the TCGA and GSE65858 cohorts were first stratified into *ATM*-high and *ATM*-low subgroups according to *ATM* mRNA expression (30 patients with the highest and lowest expression, respectively, were selected). The results showed that *ATM* expression was inversely correlated with that of *ISG15*, *IFI27*, and *OASL* in both data sets (Figure 6A–C), which was consistent with the in vitro finding that ATM inhibition elevated the expressions of these ISGs (Figure 4, Figures S1 and S2). In contrast, the expressions between the three ISGs were positively correlated with each other (Figure 6C). The demographic data of the patients, such as age, pathological stage, and survival status, were not significantly different between the high and low *ATM* subgroups (Supplementary Tables S1 and S2).

### 3.4. High-Expression of ISG15/IFI27/OASL Was Associated with the Pathways of Antigen Presentation, T-, and Natural Killer Cell-Mediated Immunity in Oral Cancer

Next, patients from the TCGA and GSE65858 cohorts were divided into two subgroups based on the joint high- and low-expression of *ISG15*/*IFI27*/*OASL* (30 patients per subgroup), and then their transcriptome data were compared by GSEA. The results showed that the high *ISG15*/*IFI27*/*OASL* subgroup exhibited several immune pathways that could favor ICB therapy, such as “CELL_KILLING”, “T_CELL_MEDIATED_CYTOTOXICITY”, “NATURAL_KILLER_CELL_MEDIATED_IMMUNITY”, “DENDRITIC_CELL_DIFFERENTIATION”, “ANTIGEN_PROCESSING_AND_PRESENTATION”, and “RESPONSE_TO_TYPE_I_INTERFERON” in TCGA (Table 2) and GSE65858 (Table 3) cohorts. The patient’s demographics, such as age, pathological stage, and survival status, were not significantly different between the high and low *ISG15*/*IFI27*/*OASL* subgroups (Supplementary Tables S3 and S4). These results suggest that high-expressions of *ISG15*/*IFI27*/*OASL* could promote immune recognition and the killing of oral cancer cells.

In contrast, these immune pathways, including “REGULATION_OF_CELL_KILLING”, “T_CELL_ACTIVATION”, “NATURAL_KILLER_CELL_MEDIATED_IMMUNITY”, “DENDRITIC_CELL_CHEMOTAXIS”, “B_CELL_ACTIVATION”, and “INTERFERON_GAMMA_PRODUCTION”, were suppressed in the cancers of *ATM*-high subgroups compared to those of ATM-low subgroups (Supplementary Tables S5 and S6). These results suggest that induction of *ISG15*/*IFI27*/*OASL* through ATM inhibition could improve suppressive TIME of oral cancer.

### 3.5. High-Expression of ISG15/IFI27/OASL Was Associated with Enriched Signatures of Plasmacytoid Dendritic Cells (pDC), CD8+ T, and NK Cells in Oral Cancer

**Figure 7:**
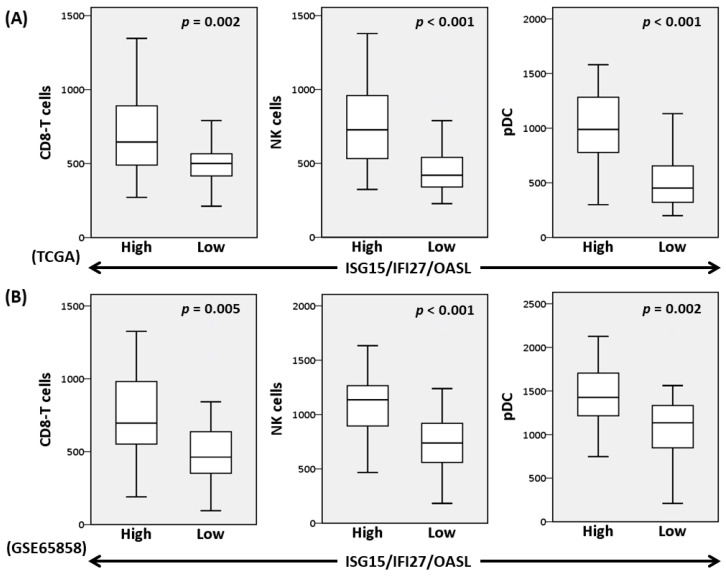
Enrichment of CD8+ T, natural killer (NK), and plasmacytoid dendritic cells (pDC) in oral cancer with high-expression of *ISG15*/*IFI27*/*OASL*. Cell-type enrichment scores in *ISG15*/*IFI27*/*OASL*-high (*n* = 30) and *ISG15*/*IFI27*/*OASL*–low (*n* = 30) subgroups of oral cancer data sets of TCGA (**A**) and GSE65858 (**B**) were analyzed using xCell algorithm. *p*, Mann–Whitney U test.

The effect of *ISG15*/*IFI27*/*OASL* expression on the configurations of tumor-infiltrated lymphocytes in oral cancer was examined using the cell-type enrichment analysis algorithm xCell [33]. The results showed that the scores of pDC, NK, and CD8+ T cells increased significantly in the high *ISG15*/*IFI27*/*OASL* subgroups of the TCGA (Figure 7A) and GSE65858 (Figure 7B) cohorts. The scores of CD4+ T cells and pro-inflammatory M1 macrophages also increased in the high *ISG15*/*IFI27*/*OASL* subgroups (Supplementary Figure S5). These results suggest that high-expressions of *ISG15*/*IFI27*/*OASL* are correlated with the increased infiltration of anticancer immune cells in oral cancer.

### 3.6. High Expressions of ISG15, IFI27, and OASL Were Associated with an Improved Response to Cisplatin in Cervical Cancer Patients

Since KU55933 induced the expressions of *ISG15*, *IFI27*, and *OASL* in CDDP-R cancer cells (Figure 4B and Figure S1), the effects of the three ISGs on the response to cisplatin therapy were examined in patients with cervical cancer from the TCGA cohort. The results demonstrated that in patients treated with cisplatin, higher expressions of the three ISGs were observed in the complete remission (CR) group compared to the progression disease (PD) group (Supplementary Table S7). However, the expression levels of the three ISGs were comparable between the groups of patients with CR and PD who did not receive cisplatin therapy (Supplementary Table S7). The demographic data of the patients, including age, survival, clinical stage, CR, and PD, with and without cisplatin therapy, are shown in Supplementary Table S8. These results suggest that the expressions of *ISG15*, *IFI27*, and *OASL* are also correlated with the clinical response to cisplatin therapy.

## 4. Discussion

Current ICB therapy includes patients with cisplatin-refractory cancers; however, the response rates require improvement. This study demonstrated that ATMi was able to induce the interferon response and elevate the expression of ISGs in both CDDP-R and parental cancer cells (Figure 2 and Figure 4, Figures S1 and S2). In addition to human cancer cells, ATM inhibition also activates the interferon response in Drosophila glial cells and causes neurodegeneration in the fly, which recapitulates the phenotype of human ataxia telangiectasia (AT) caused by *ATM* gene mutation [34,35]. Moreover, activation of the interferon response is observed in fibroblasts isolated from AT patients, and sera from AT patients can inhibit virus infection [36]. These results suggest that the activation of the interferon response is a general consequence of ATM inhibition.

The ATM inhibition-induced interferon response can improve ICB therapy through sculpting the “hot” TIME in animal models [22,24]. On the other hand, because ATM can stimulate the differentiation of myofibroblastic cancer-associated fibroblasts, which promote suppressive TIME, ATM inhibition in fibroblasts can increase tumor-infiltrating CD8+ T cells and improve ICB therapy in mouse models of lung and colon cancers [37]. Furthermore, Liu et al. demonstrated that KU55933 can prevent tumor- or regulatory T cell-induced effector T cell senescence and enhance antitumor immunity in a mouse model of melanoma [38]. ATM inhibition can also enhance radiation-induced inflammatory signaling and cancer cell death [39]. Together, these studies suggest that ATM inhibition is an attractive strategy to induce the antitumor immune response in the TIME and improve the efficacy of ICB therapy.

Regarding ATM inhibition in clinical settings, cancer patients with *ATM* gene mutation have benefited from ICB therapy compared to those with wild-type *ATM* gene [22,25,27,40,41,42], because *ATM* gene mutation is found to be correlated with TMB-H, dMMR, or the increased expression of PD-L1 [42,43], which are known biomarkers for responses to ICB therapy [9,10,11,12]. Furthermore, increased numbers of cytotoxic CD8+ T cells and activated DCs in TIME are observed in cancers with *ATM* gene mutations [22,25,40,41]. However, the influence of gene mutation in *ISG15*, *IFI27*, and *OASL*, which are induced by ATM inhibition, on the TIME and ICB therapy is unclear and awaits future research.

On the other hand, low *ATM* expression is found to be correlated with increased tumor-infiltrating CD8+ T cells in breast cancer and fumarate hydratase-deficient renal cell carcinoma [44,45]. However, patients with breast cancer without ICB therapy and with low *ATM* expression exhibit lower OS rates than those with high *ATM* expression [45]. We also reported that patients with head and neck cancer without ICB therapy and with low *ATM* expression in their tumors exhibited poor OS rates [29]. Therefore, the benefit of low *ATM* expression may be limited to patients who received ICB therapy. In this regard, patients with low *ATM* expression are recommended to receive ICB therapy instead of the previously established standard therapies.

Although ATM inhibition is closely correlated with the active immune response in the TIME, the roles of ATM inhibition-induced ISGs in ICB therapy have been less explored. In this study, the effects of KU55933-induced ISGs on ICB therapy were evaluated with clinical data, which showed a benefit of high ISG expressions on patients’ OS rates (Figure 5 and Figure S4). In oral cancer, high expressions of *ISG15*, *IFI27*, and *OASL* were found to be associated with low *ATM* expression (mimicry of ATM inhibition), the activation of inflamed immune pathways, and elevated tumor-infiltrated scores of pDC, NK, and CD8+ T cells (Figure 6 and Figure 7, Table 1 and Table 2). These results are correlated with the fact that ISG15 is able to induce interferon-γ production, promote NK cell proliferation, and stimulate DC maturation [46,47,48,49]. ISG15 can serve as a vaccine adjuvant to enhance human papillomavirus E7-specific interferon-γ and CD8+ T-cell responses [50]. ISG15 is also involved in Listeria-based vaccine-induced interferon-γ and CD8+ T-cell responses in mouse models of renal cell carcinoma [51]. In melanoma patients treated with anti-PD1 and cetirizine combination therapy, upregulations of *IFI27*, *IFIT1*, and *IFIT3* are associated with M1 polarization of macrophages and improved patient outcome [52]. These data are indicative of the contributions of ISG15 and IFI27 to ICB therapy. However, additional investigations using in vitro cell and animal models are needed to illustrate the underlying mechanism of ATM inhibition-induced ISGs in response to ICB therapy. Moreover, altered epigenetic regulation also contributes to ISG expression, the interferon response, and ICB therapy, which requires further research [53,54].

This study also found that high expressions of *ISG15*, *IFI27*, and *OASL* were associated with complete remission in patients with cervical cancer treated with cisplatin but not in those without cisplatin-based therapy (Supplementary Table S7). In this regard, ISG15 has been reported to inhibit *ABCC2* expression and re-sensitize CDDP-R ovarian cancer cells [55]. Moreover, *ISG15* is found to be downregulated in CDDP-R ovarian cancer [56]. However, the roles of IFI27 and OASL in CDDP-R cancer cells await future investigation.

Another limitation of the present study is the lack of clinical response data for ICB therapy in patients with oral cancer. Several clinical trials of ICB therapy include oral cancer patients with genomic analysis data [57,58]. Once these results are available to the public, the roles of ATMi-induced ISGs in these patients can be evaluated. Based on the published results so far, interferon-γ and T cell-inflamed gene signatures, TMB, and PD-L1 expression are correlated with the response to ICB therapy in patients with head and neck cancer [59,60]. These results are indicative of an inflamed immunophenotype, which is similar to the findings of the present study.

## 5. Conclusions

ATM inhibitors can induce interferon responses and have the potential to enhance the efficacy of ICB therapy in cancer patients, including those with cisplatin-refractory cancers. ISG expressions, such as *ISG15*, *IFI27*, and *OASL*, may serve as biomarkers to guide patient selection for ICB therapy; however, more clinical investigations are required to validate their biomarker roles in ICB therapy.

## Figures and Tables

**Table 1:** Primer sequences for quantitative PCR.

Gene	Forward Primer (5′ to 3′)	Reverse Primer (5′ to 3′)
*ISG15*	ACAGCCATGGGCTGGGA	CCTTCAGCTCTGACACCGAC
*IFI27*	CGGTGAGGTCAGCTTCACAT	CCTGCTCGGGTTAATTCCGT
*OASL*	GCCAACTAAGCTGAAGAGCC	GCATAGAGAGGGGGCAGATTG
*ISG20*	ACACGTCCACTGACAGGCTGTT	ATCTTCCACCGAGCTGTGTCCA
*DDX58*	CACCTCAGTTGCTGATGAAGGC	GTCAGAAGGAAGCACTTGCTACC
*DDX60*	GGCTTCCAAGGGAGATGACC	TGTCCATGACTCTGGGTTGC
*CCL5*	CCAGCAGTCGTCTTTGTCAC	CTCTGGGTTGGCACACACTT
*IFIT3*	AGAAAAGGTGACCTAGACAAAGC	CCTTGTAGCAGCACCCAATCT
*GAPDH*	AGCCACATCGCTCAGACAC	GCCCAATACGACCAAATCC

**Table 2:** Activated immune pathways in oral cancer with high-expression of *ISG15*/*IFI27*/*OASL* (TCGA).

NAME (TCGA)	SIZE	NES	NOM-p	FDR-q
GOBP_INTERFERON_GAMMA_PRODUCTION	116	3.130	<0.001	<0.001
GOBP_RESPONSE_TO_TYPE_I_INTERFERON	82	2.929	<0.001	<0.001
GOBP_CELL_KILLING	185	3.276	<0.001	<0.001
GOBP_POSITIVE_REGULATION_OF_CELL_KILLING	86	3.085	<0.001	<0.001
GOBP_NATURAL_KILLER_CELL_MEDIATED_IMMUNITY	76	3.118	<0.001	<0.001
GOBP_NATURAL_KILLER_CELL_ACTIVATION	97	2.870	<0.001	<0.001
GOBP_T_CELL_MEDIATED_IMMUNITY	176	3.005	<0.001	<0.001
GOBP_T_CELL_MEDIATED_CYTOTOXICITY	51	2.755	<0.001	<0.001
GOBP_DENDRITIC_CELL_DIFFERENTIATION	48	2.424	<0.001	<0.001
GOBP_DENDRITIC_CELL_CYTOKINE_PRODUCTION	18	1.835	<0.001	<0.001
GOBP_ANTIGEN_PROCESSING_AND_PRESENTATION_OF_PEPTIDE_ANTIGEN	104	2.972	<0.001	<0.001
GOBP_ANTIGEN_PROCESSING_AND_PRESENTATION	65	2.965	<0.001	<0.001

**Table 3:** Activated immune pathways in oral cancer with high-expression of *ISG15*/*IFI27*/*OASL* (GSE65858).

NAME (GSE65858)	SIZE	NES	NOM-p	FDR-q
GOBP_RESPONSE_TO_TYPE_I_INTERFERON	66	2.508	<0.001	<0.001
GOBP_RESPONSE_TO_INTERFERON_GAMMA	123	2.266	<0.001	<0.001
GOBP_CELL_KILLING	155	2.221	<0.001	<0.001
GOBP_LEUKOCYTE_MEDIATED_CYTOTOXICITY	117	2.081	<0.001	<0.001
GOBP_NATURAL_KILLER_CELL_MEDIATED_IMMUNITY	68	2.032	<0.001	<0.001
GOBP_REGULATION_OF_NATURAL_KILLER_CELL_ACTIVATION	37	1.747	0.005	0.024
GOBP_REGULATION_OF_T_CELL_MEDIATED_IMMUNITY	83	2.231	<0.001	<0.001
GOBP_REGULATION_OF_T_CELL_MEDIATED_CYTOTOXICITY	38	2.080	<0.001	<0.001
GOBP_DENDRITIC_CELL_DIFFERENTIATION	45	1.890	0.002	0.005
GOBP_DENDRITIC_CELL_CYTOKINE_PRODUCTION	17	1.707	0.009	0.035
GOBP_ANTIGEN_PROCESSING_AND_PRESENTATION_OF_PEPTIDE_ANTIGEN	61	2.470	<0.001	<0.001
GOBP_ANTIGEN_PROCESSING_AND_PRESENTATION	105	2.342	<0.001	<0.001

## Data Availability

The transcriptome data sets GSE178115, GSE21656 (accessed on 26 July 2022), and GSE65858 (accessed on 21 March 2021) are available in the NCBI GEO database (https://www.ncbi.nlm.nih.gov/geo/). Clinical and transcriptome data of oral (HNSC, accessed on 15 July 2020) and cervical (CESC, accessed on 14 June 2022) cancer cohorts of TCGA can be downloaded from the Genomic Data Commons Data Portal (https://portal.gdc.cancer.gov/) or UCSC Xena (https://xenabrowser.net/). Additional data in this study are available from the corresponding authors upon reasonable request.

## Supplementary Materials

The following supporting information can be downloaded at https://www.mdpi.com/article/10.3390/cells12091288/s1, Figure S1: Upregulation of ISG15, IFI27, and OASL gene expression by KU55933 (KU) in parent (CAL27) and cisplatin-resistant (CDDP-R, CAL-A8) oral cancer cells was demonstrated by RT-qPCR; Figure S2: Upregulation of interferon-stimulated genes by KU55933 in cisplatin-resistant cancer cells was demonstrated by RT-qPCR; Figure S3: Downregulation of interferon-stimulated genes in cisplatin-resistant cancer cells compared to parent cancer cells; Figure S4: Kaplan–Meier plots of overall survival of cancer patients who received immune checkpoint blockade therapy; Figure S5: Enrichment of CD4+ T cells and M1 macrophages in oral cancer with high expression of ISG15/IFI27/OASL; Table S1: The patient demographics of TCGA cohort based on ATM expression; Table S2: The patient demographics of GSE65858 cohort based on ATM expression; Table S3: The patient demographics of TCGA cohort based on ISG expression; Table S4: The patient demographics of GSE65858 cohort based on ISG expression; Table S5: The significantly downregulated immune pathways in ATM-high subgroup of TCGA cohort; Table S6: The significantly downregulated immune pathways in ATM-high subgroup of GSE65858 cohort; Table S7: Expression of interferon-stimulated genes in cervical cancer patients with or without cisplatin therapy; Table S8: The patient demographics of TCGA CESC cohort.
