# Supplementary material for: ATM Inhibition-Induced ISG15/IFI27/OASL Is Correlated with Immunotherapy Response and Inflamed Immunophenotype
Source: Cells. 2023 Apr 30;12(9):1288. doi: 10.3390/cells12091288 (PMC10177353; doi:10.3390/cells12091288)
Supplement: Supplementary file 1 [file cells-12-01288-s001.zip › cells-2349596-supplementary.pdf]

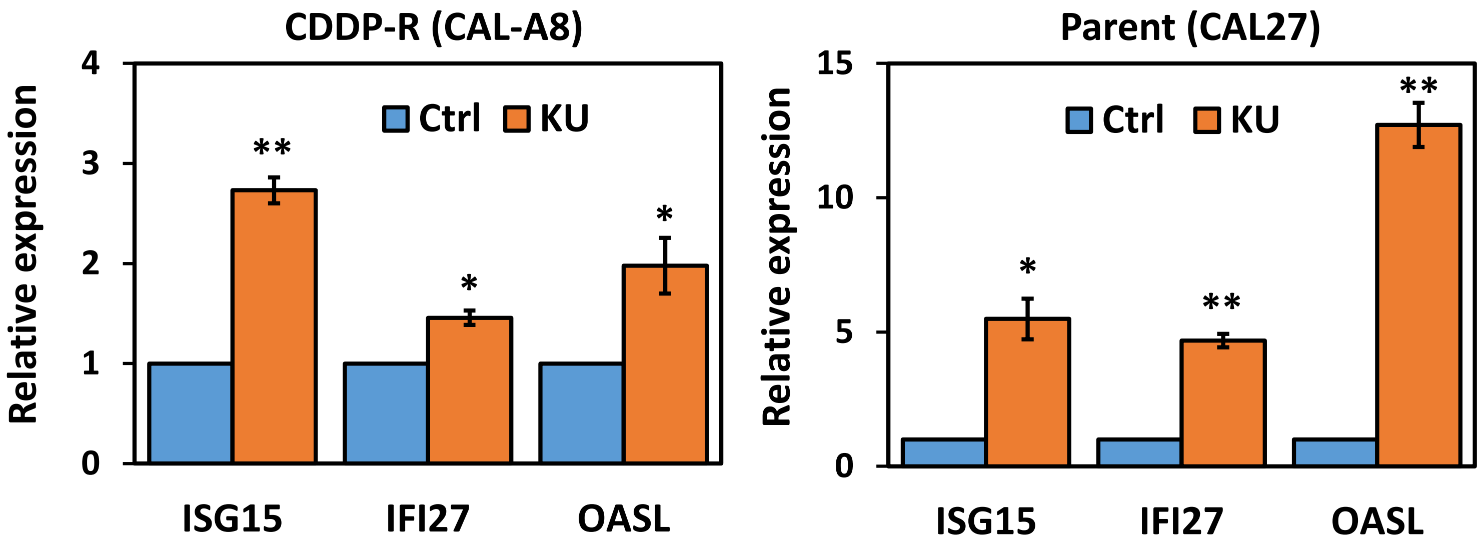

Figure S1. Upregulation of ISG15, IFI27, and OASL gene expression by KU55933 (KU) in parent (CAL27) and cisplatin-resistant (CDDP-R, CAL-A8) oral cancer cells was demonstrated by RT-qPCR. *, $p < 0.05$ versus control (Ctrl). **, $p < 0.001$ versus control (Ctrl).

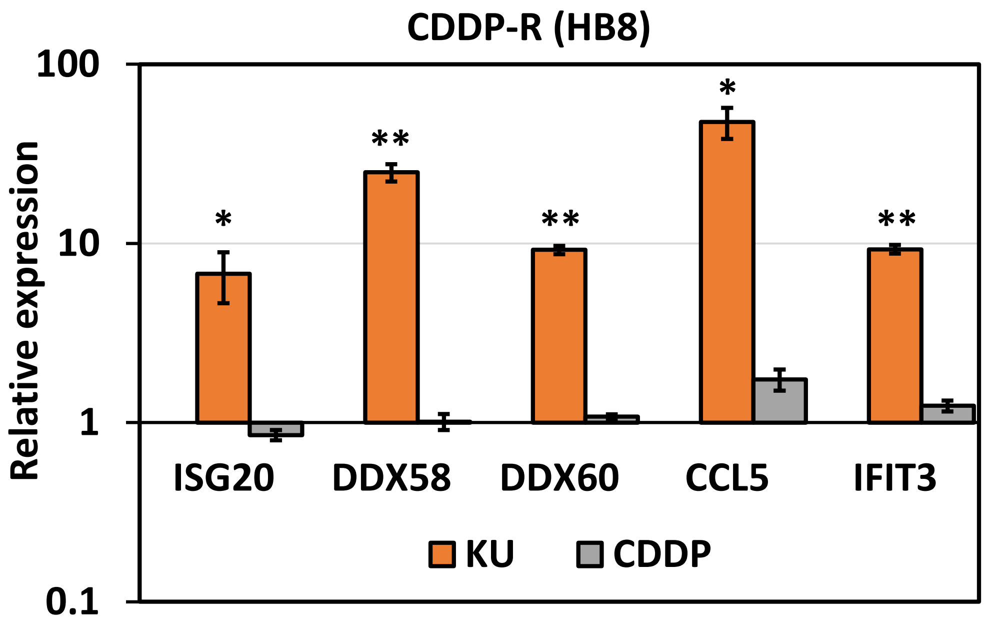

Figure S2. Upregulation of interferon-stimulated genes by KU55933 in cisplatin-resistant cancer cells was demonstrated by RT-qPCR. CDDP-R, cisplatin-resistant. KU, KU55933. CDDP, cisplatin. *, $p < 0.05$ versus control. **, $p < 0.001$ versus control.

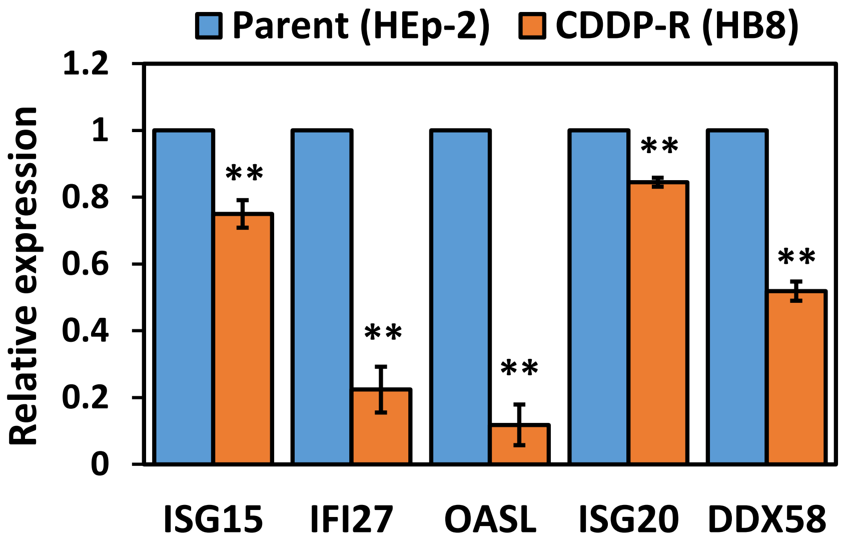

Figure S3. Downregulation of interferon-stimulated genes in cisplatin-resistant cancer cells compared to parent cancer cells. The gene expression levels were determined by RT-qPCR. CDDP-R, cisplatin-resistant. **, $p < 0.001$ versus parent cells (HEp-2).

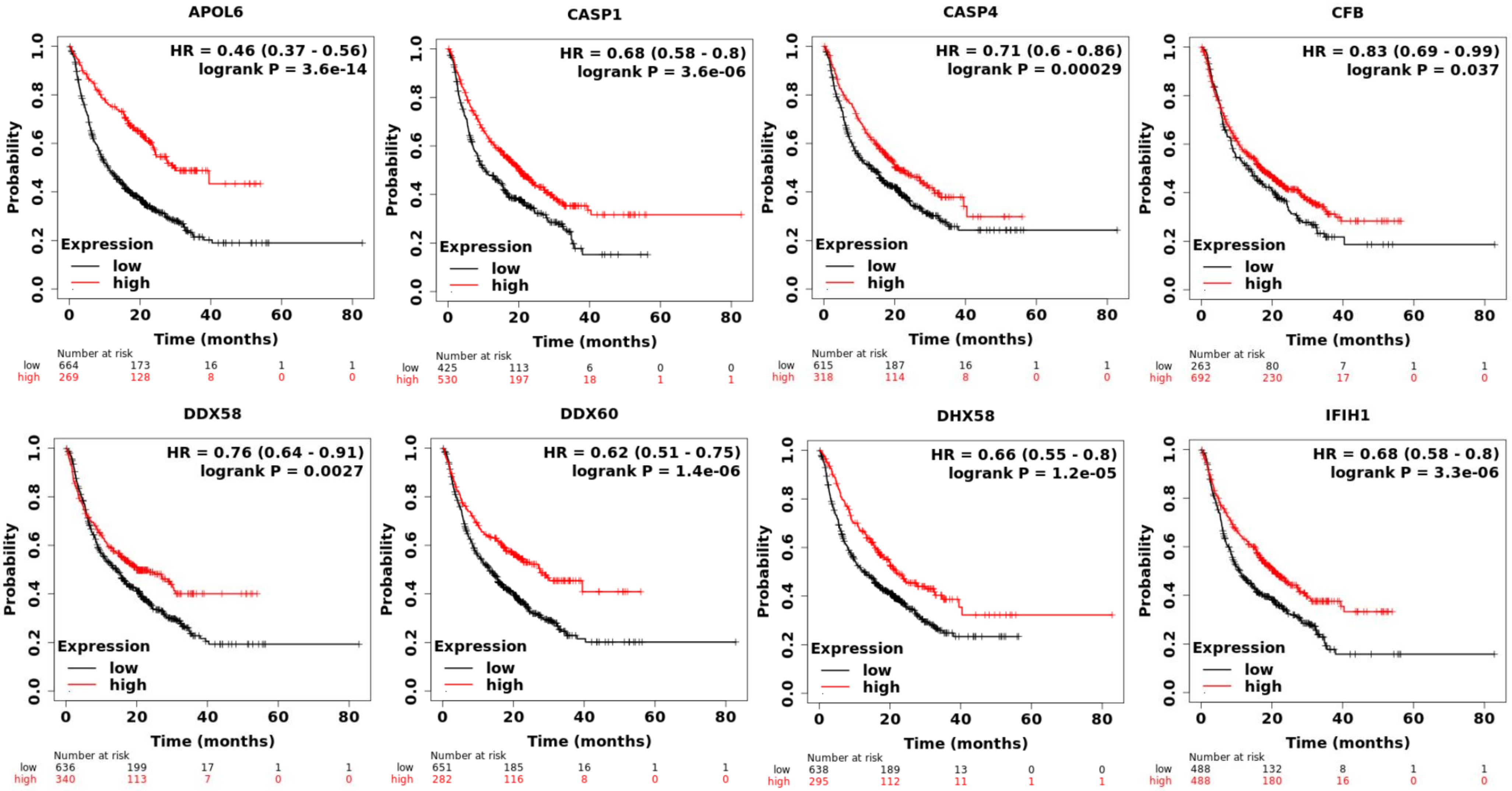

Figure S4. Kaplan-Meier plots of overall survival of cancer patients who received immune checkpoint blockade therapy. The best cut-off values were determined by ROC curve analysis.

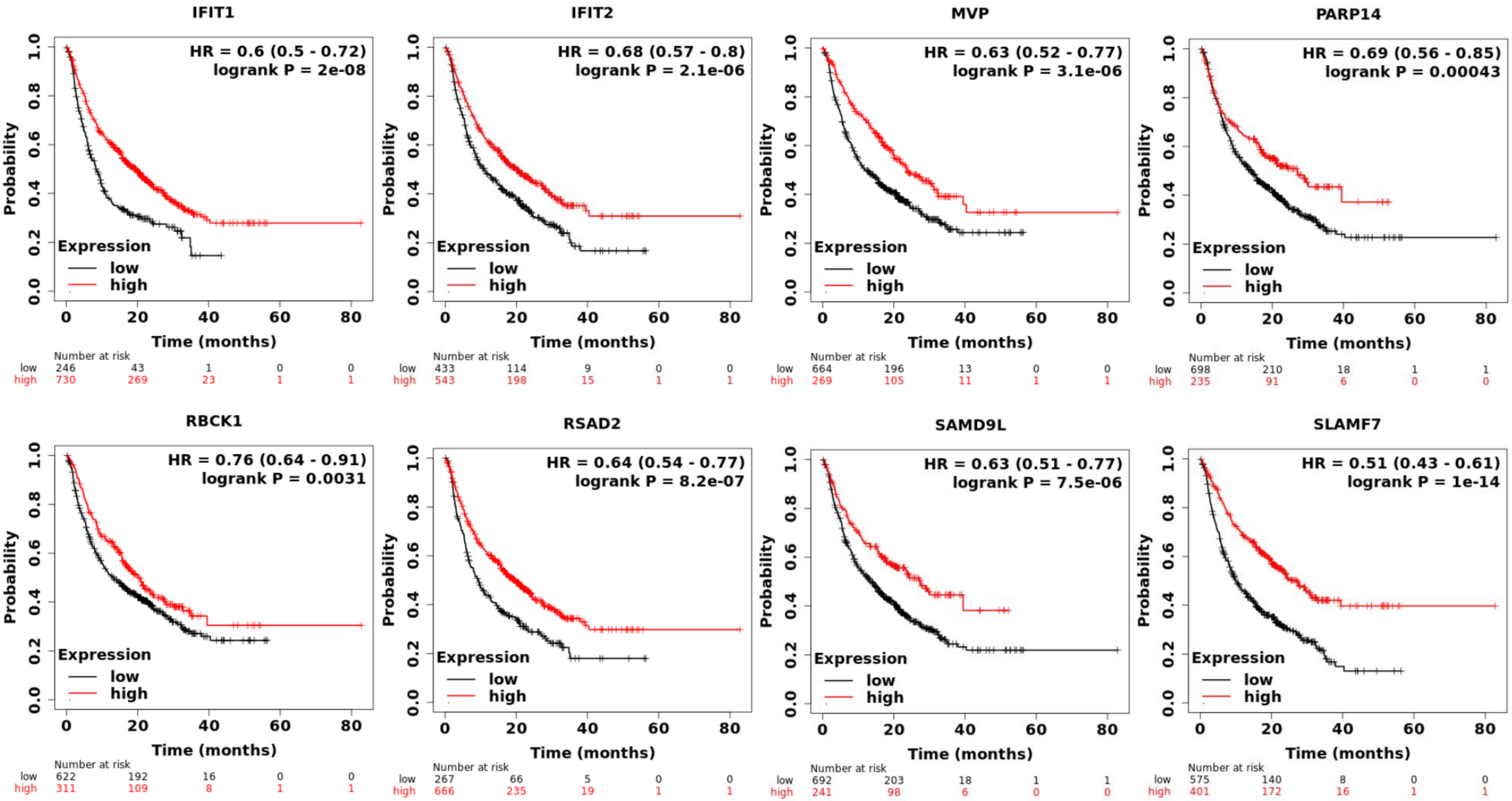

Figure S4. (Cont.) Kaplan-Meier plots of overall survival of cancer patients who received immune checkpoint blockade therapy. The best cut-off values were determined by ROC curve analysis.

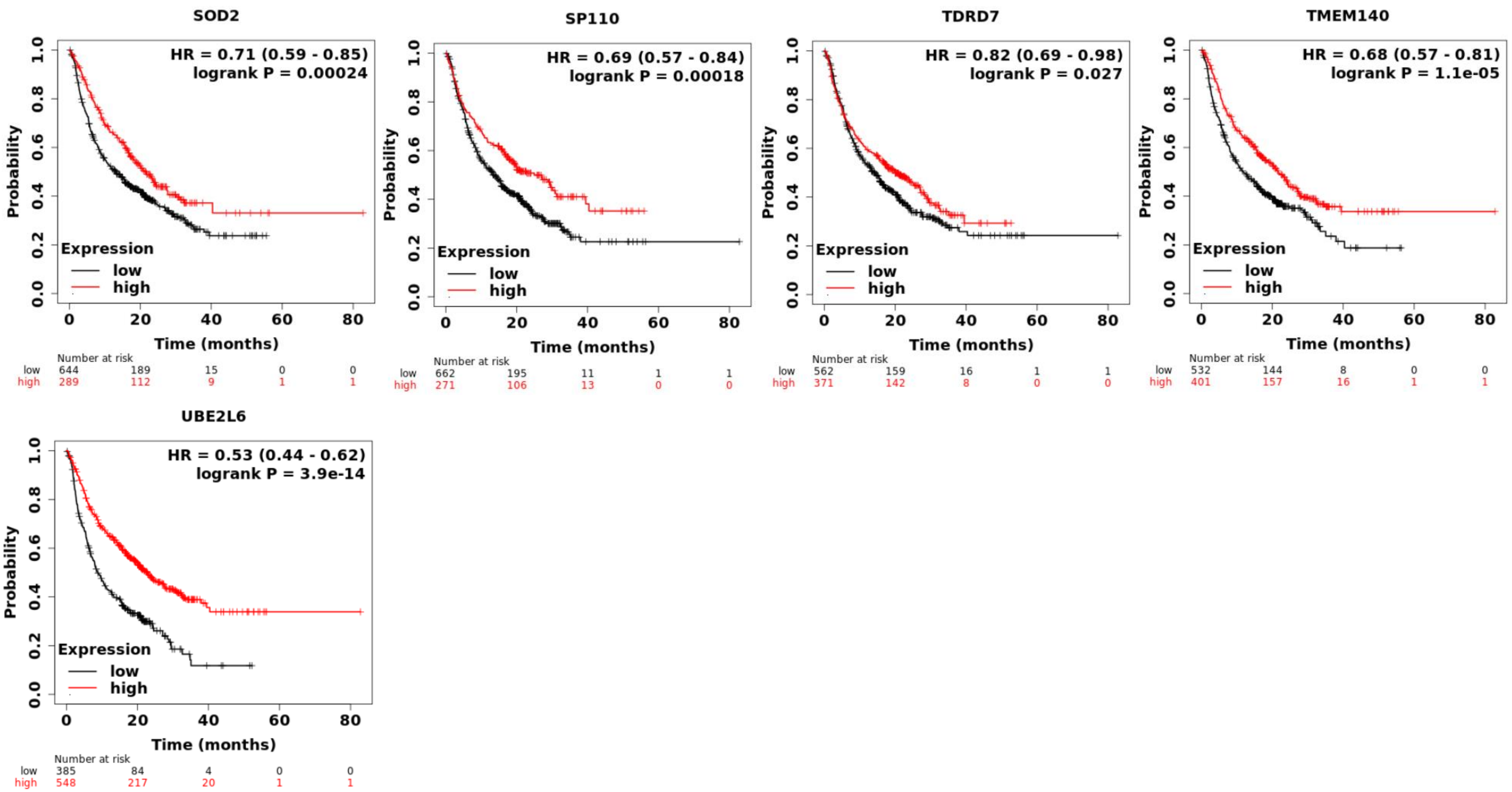

Figure S4. (Cont.) Kaplan-Meier plots of overall survival of cancer patients who received immune checkpoint blockade therapy. The best cut-off values were determined by ROC curve analysis.

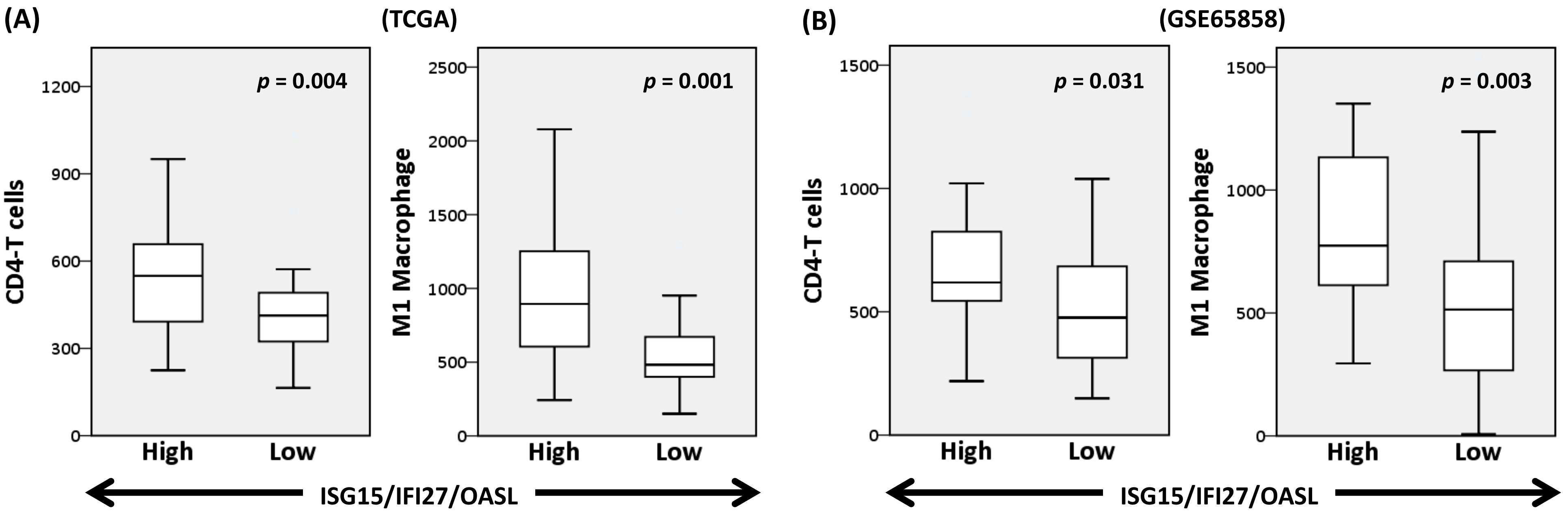

Figure S5. Enrichment of CD4+ T cells and M1 macrophages in oral cancer with high expression of ISG15/IFI27/OASL. Cell type enrichment scores in ISG15/IFI27/OASL-High (n=30) and ISG15/IFI27/OASL–Low (n=30) subgroups of TCGA (A) and GSE65858 (B) oral cancer data sets were analyzed using xCell algorithm.

Table S1. The patient demographics of TCGA cohort based on ATM expression.

Characteristics	ATM-High (n=30)	ATM-Low (n=30)	*p*
Age (Years) [a]	58 (49, 66)	58 (53, 65)	0.981
Male (%)	20 (66.6)	20 (66.6)	0.695
Survival status: Living (%)	16 (53.3)	16 (57.1)	0.771
OS [b] (Days) [a]	593 (375, 873)	527 (295, 1079)	0.950
PFS [c] (Days) [a]	568 (431, 662)	546 (219, 1370)	0.941
Tumor stage (2 missing)			
Stage I (%)	3 (10.0)	0 (0)	0.084
Stage II (%)	3 (10.0)	4 (13.3)	
Stage III (%)	10 (33.3)	4 (13.3)	
Stage VI (%)	12 (40.0)	14 (46.7)	
ATM [a]	10.3 (10.2, 10.8)	7.6 (7.3, 7.8)	<0.001
OASL [a]	8.8 (7.7, 10.4)	10.5 (9.88, 11.5)	<0.001
IFI27 [a]	13.1 (12.3, 13.4)	15.0 (14.5, 15.8)	<0.001
ISG15 [a]	11.4 (9.9, 12.2)	14.2 (12.6, 15.3)	<0.001

[a] median (interquartile range). [b] overall survival. [c] progression free survival.

Table S2. The patient demographics of GSE65858 cohort based on ATM expression.

Characteristics	ATM-High (n=30)	ATM-Low (n=30)	*p*
Age (Years) [a]	56 (48, 70)	57 (52, 68)	0.701
Male (%)	24 (80)	24 (80)	>0.999
Survival status: Living (%)	21 (70)	21 (70)	>0.999
OS [b] (Days) [a]	849 (667, 1016)	787 (581, 982)	0.589
PFS [c] (Days) [a]	806 (380, 1011)	691 (334, 957)	0.482
Tumor stage			
Stage I (%)	4 (13.3)	4 (13.3)	0.534
Stage II (%)	4 (13.3)	4 (13.3)	
Stage III (%)	0 (0)	3 (10.0)	
Stage VI (%)	22 (73.3)	19 (63.3)	
ATM [a]	7.9 (7.8, 8.1)	7.4 (7.3, 7.5)	<0.001
OASL [a]	7.8 (6.9, 8.4)	8.3 (7.6, 8.9)	0.001
IFI27 [a]	11.6 (10.5, 12.2)	12.3 (11.6, 12.5)	0.003
ISG15 [a]	9.8 (8.7, 11.0)	10.7 (9.8, 11.6)	0.028

[a] median (interquartile range). [b] overall survival. [c] progression free survival.

Table S3. The patient demographics of TCGA cohort based on ISG [a] expression.

Characteristics	ISG-Low (n=30)	ISG-High (n=30)	*p*
Age (Years) [b]	66 (54, 72)	56 (50, 69)	0.071
Male (%)	25 (86.2)	17 (59.0)	0.019
Survival status: Living (%)	16 (57.1)	20 (69.0)	0.355
OS [c] (Days) [b]	518.5 (385, 939)	428 (225, 1043)	0.508
PFS [d] (Days) [b]	394 (185, 783)	342 (210, 698)	0.920
Tumor stage (2 missing)			
Stage I (%)	2 (8.3)	2 (8.3)	0.495
Stage II (%)	5 (20.8)	2 (8.3)	
Stage III (%)	4 (16.7)	9 (37.5)	
Stage VI (%)	13 (54.2)	11 (45.9)	
ATM [b]	9.53 (8.9, 9.8)	8.85 (7.8, 9.2)	<0.001
OASL [b]	6.55 (5.2, 6.9)	10.48 (10.3, 10.8)	<0.001
IFI27 [b]	11.17 (9.5, 12.0)	14.48 (13.4, 14.9)	<0.001
ISG15 [b]	9.06 (8.4, 10.1)	13.13 (12.5, 13.8)	<0.001

[a] ISG15/IFI27/OASL. [b] median (interquartile range). [c] overall survival. [d] progression free survival.

Table S4. The patient demographics of GSE65858 cohort based on ISG [a] expression.

Characteristics	ISG-Low (n=30)	ISG-High (n=30)	*p*
Age (Years) [b]	54 (48, 71)	58 (51, 71)	0.220
Male (%)	25 (83.3)	23 (76.7)	0.519
Survival status: Living (%)	21 (70)	20 (66.7)	0.780
OS [c] (Days) [b]	825 (596, 967)	845 (664, 1064)	0.340
PFS [d] (Days) [b]	757.5 (372, 967)	790 (563, 1027)	0.370
Tumor stage (2 missing)			
Stage I (%)	5 (16.7)	4 (13.3)	0.717
Stage II (%)	5 (16.7)	4 (13.3)	
Stage III (%)	2 (6.7)	4 (13.3)	
Stage VI (%)	18 (60.0)	18 (60.0)	
ATM [b]	7.7 (7.5, 8.0)	7.5 (7.4, 7.7)	<0.001
OASL [b]	7.1 (6.8, 7.4)	8.8 (8.4, 9.2)	0.008
IFI27 [b]	10.8 (10.0, 11.3)	12.5 (12.3, 12.7)	0.005
ISG15 [b]	9.0 (8.3, 9.4)	11.4 (10.8, 11.7)	0.023

[a] ISG15/IFI27/OASL. [b] median (interquartile range). [c] overall survival. [d] progression free survival.

Table S5. The significantly downregulated immune pathways in ATM-High subgroup of TCGA cohort.

TCGA_ATM (High vs. Low)	SIZE	NES	NOM-p	FDR-q
GOBP_INTERFERON_GAMMA_PRODUCTION	116	-1.468	0.001	0.098
GOBP_INTERFERON_BETA_PRODUCTION	56	-1.413	0.021	0.126
GOBP_POSITIVE_REGULATION_OF_CELL_KILLING	65	-1.637	<0.001	0.039
GOBP_REGULATION_OF_CELL_KILLING	104	-1.592	<0.001	0.049
GOBP_POSITIVE_REGULATION_OF_NATURAL_KILLER_CELL_MEDIATED_CYTOTOXICITY	26	-1.555	0.012	0.061
GOBP_REGULATION_OF_NATURAL_KILLER_CELL_MEDIATED_IMMUNITY	50	-1.436	0.019	0.113
GOBP_ALPHA_BETA_T_CELL_ACTIVATION	169	-1.585	<0.001	0.051
GOBP_ALPHA_BETA_T_CELL_DIFFERENTIATION	116	-1.597	<0.001	0.048
GOBP_DENDRITIC_CELL_CHEMOTAXIS	27	-1.773	<0.001	0.013
GOBP_DENDRITIC_CELL_MIGRATION	34	-1.871	0.001	0.004
GOBP_B_CELL_PROLIFERATION	103	-1.845	<0.001	0.005
GOBP_B_CELL_ACTIVATION	275	-1.620	<0.001	0.042

Table S6. The significantly downregulated immune pathways in ATM-High subgroup of GSE65858 cohort.

GSE65858_ATM (High vs. Low)	SIZE	NES	NOM-p	FDR-q
GOBP_CELLULAR_RESPONSE_TO_INTERFERON_GAMMA	104	-1.767	<0.001	0.020
GOBP_INTERFERON_GAMMA_PRODUCTION	105	-1.524	0.005	0.137
GOBP_REGULATION_OF_LEUKOCYTE_MEDIATED_CYTOTOXICITY	77	-1.846	<0.001	0.008
GOBP_REGULATION_OF_CELL_KILLING	91	-1.826	<0.001	0.010
GOBP_NATURAL_KILLER_CELL_ACTIVATION	77	-1.777	<0.001	0.018
GOBP_NATURAL_KILLER_CELL_MEDIATED_IMMUNITY	68	-1.578	0.005	0.102
GOBP_ALPHA_BETA_T_CELL_ACTIVATION	157	-2.192	<0.001	<0.001
GOBP_ALPHA_BETA_T_CELL_PROLIFERATION	43	-2.133	<0.001	<0.001
GOBP_DENDRITIC_CELL_ANTIGEN_PROCESSING_AND_PRESENTATION	15	-2.047	<0.001	<0.001
GOBP_DENDRITIC_CELL_CHEMOTAXIS	26	-1.860	0.002	0.006
GOBP_B_CELL_ACTIVATION	82	-2.422	<0.001	<0.001
GOBP_B_CELL_PROLIFERATION	19	-2.420	<0.001	<0.001

Table S7. Expression of interferon-stimulated genes in cervical cancer patients with or without cisplatin therapy.

		PD/SD [a] [mean (S.D.)] [b]	CR [c] [mean (S.D.)]	*p* [d]
ISG15	w CDDP [e]	10.744 (1.447)	12.560 (1.354)	0.002
	wo CDDP [f]	11.086 (2.001)	11.296 (1.568)	0.522
IFI27	w CDDP	12.875 (0.944)	13.720 (0.876)	0.010
	wo CDDP	12.588 (2.088)	12.916 (1.394)	0.944
OASL	w CDDP	8.274 (1.261)	10.022 (1.271)	0.001
	wo CDDP	8.726 (2.324)	8.703 (1.617)	0.912

[a] progression disease/stable disease. [b] Gene expression (RSEM) shown as mean and standard deviation (S.D.). [c] complete remission. [d] Mann-Whitney U test. [e] with cisplatin therapy (11 PD, 42 CR). [f] without cisplatin therapy (11 PD/SD, 96 CR).

Table S8. The patient demographics of TCGA CESC [a] cohort.

	With Cisplatin Therapy		Without Cisplatin Therapy	
	PD [b] (n=11)	CR [c] (n=42)	PD/SD [d] (n=11)	CR (n=96)
Age (Years) [e]	46 (37, 51)	43 (37, 54)	45 (32, 56)	45 (37, 53)
Survival status: Living (%)	2 (18.2)	39 (92.9)	3 (27.3)	91 (94.8)
Overall survival (Days) [e]	370 (292, 581)	949 (601, 1669)	634 (359, 1048)	799 (491, 1376)
Clinical stage				
Stage I (%)	1 (9.1)	15 (36.6)	5 (45.5)	66 (68.8)
Stage II (%)	4 (36.4)	17 (41.5)	3 (27.3)	17 (17.7)
Stage III (%)	2 (18.2)	7 (17.1)	2 (18.2)	12 (12.5)
Stage VI (%)	4 (36.4)	2 (4.9)	1 (9.1)	1 (1.0)

[a] cervical squamous cell carcinoma and endocervical adenocarcinoma. [b] progression disease. [c] complete remission. [d] progression disease/stable disease. [e] median (interquartile range).
